# Effects of β-caryophyllene and oxygen availability on cholesterol and fatty acids in breast cancer cells

**DOI:** 10.1371/journal.pone.0281396

**Published:** 2023-03-09

**Authors:** Christopher J. Frost, Andrea Ramirez-Mata, Ram B. Khattri, Matthew E. Merritt, Susan C. Frost

**Affiliations:** 1 BIO5 Institute, University of Arizona, Tucson, AZ, United States of America; 2 Department of Biology, University of Louisville, Louisville, KY, United States of America; 3 Department of Biochemistry and Molecular Biology, University of Florida, Gainesville, FL, United States of America; Université Clermont Auvergne - Faculté de Biologie, FRANCE

## Abstract

Hypoxia is a common feature of most solid tumors, one that favors tumor progression and limits treatment effectiveness. Targeting hypoxia has long been a goal in cancer therapy, by identifying factors that reverse or ameliorate the effects of hypoxia on cancer cells. We, and others, have shown that β-caryophyllene (BCP) exhibits anti-proliferative properties in cancer cells. We have further shown that non-cytotoxic concentrations of BCP affect cholesterol and lipid biosynthesis in hypoxic hBrC cells at both transcriptional and translational levels. This led us to hypothesize that BCP may reverse the hypoxic phenotype in hBrC cells. To test this, we determined the effect of BCP on hypoxic sensitive pathways, including oxygen consumption, glycolysis, oxidative stress, cholesterol and fatty acid biosynthesis, and ERK activation. While each of these studies revealed new information on the regulation by hypoxia and BCP, only the lipidomic studies showed reversal of hypoxic-dependent effects by BCP. These later studies showed that hypoxia-treated samples lowered monounsaturated fatty acid levels, shifting the saturation ratios of the fatty acid pools. This signature was ameliorated by sub-lethal concentrations of BCP, possibly through an effect on the C:16 fatty acid saturation ratios. This is consistent with BCP-induced upregulation of the stearoyl-CoA desaturase (SCD) gene, observed previously. This suggests that BCP may interfere with the lipid signature modulated by hypoxia which could have consequences for membrane biosynthesis or composition, both of which are important for cell replication.

## Introduction

While substantial progress has been made towards treating breast cancer, such as surgical resections or adjuvant therapies, highly aggressive cancers (i.e., invasive) remain a challenge. In 2022, the American Cancer Society (ACS) predicted that nearly 280,000 women will be newly diagnosed with invasive breast cancer, along with 51,400 cases of ductal carcinoma *in situ* (DCIS) [1]. The ACS further estimates that 43,250 women will die from this disease during this year. This translates to one death every 12 minutes. While the overall death rates from breast cancer have dropped by 42% since 1989, primarily because of improved screening, invasive breast cancer has increased by 0.5% each year since the mid 2000’s. One of the deadliest types of breast cancers in women are those with the triple negative phenotype (TNBC), where expression of the estrogen, progesterone, and HER2 receptors is either low or absent [2]. This type of cancer is one of the most difficult to treat due to lack of targeted therapies and its inherent therapeutic resistance.

One of the initiating events in cellular transformation is the repeated exposure to hypoxia, which selects for cells with mutations that constitutively upregulate glycolysis [3], coined the “glycolytic phenotype”. This is clinically relevant as increased glucose uptake is observed in FdG-PET scans of the vast majority of breast cancers that are sufficiently large to image. In addition to glycolytic upregulation, lactate dehydrogenase (LDH) is induced, diverting glucose carbons from the mitochondrial oxidative pathway even in the presence of oxygen. This “aerobic” glycolysis was originally described by Warburg nearly 100 years ago [4, 5] which contributes, in part, to the acidification of the tumor microenvironment [6–9] and induces apoptosis in normal cells [10]. At the same time, these same conditions allow the cancer cell population to expand [11, 12]. We now know that the glycolytic phenotype is primarily mediated by the stabilization of the HIF1α transcription factor at low oxygen [13], which then binds to the promoter regions of the genes that encode for select glucose transporters, glycolytic enzymes, and auxiliary proteins that regulate glycolytic flux.

Plants produce a remarkable array of complex chemicals beyond those that are required for their own growth, reproduction, or protection [14]. These “specialized” metabolites have been used in the treatment of human diseases for generations, even when their active components and molecular targets are not well-defined [15]. While specialized metabolites are derived from a number of biosynthetic pathways, terpenes (like Paclitaxel, one the most successful cytotoxic drugs in treating breast cancer [16]) are the most abundant class of plant secondary metabolites [17]. Over the last decade, we and several other investigators have demonstrated that another terpene, β-caryophyllene (BCP), exhibits anti-proliferative properties in cancer cells [15, 18–24], although our work uniquely addressed the role of hypoxia as a confounding factor [24]. (For a comprehensive review of the chemopreventive potential of BCP, see Di Sotto et al. [25]). BCP is a bicyclic sesquiterpene and found in many essential oils including that of *Eugenia caryophyllata* (clove), which contains caryophyllene in sufficiently high quantities that it is often used as a source for the isolation of the compound [26]. Because of its pleasant aroma, BCP is used in a number of products for flavor or fragrance. BCP is also on the list of food additives approved by the United States, Food and Drug Administration and further evaluated by FEMA (Flavor and Extract Manufacturers Association) to achieve GRAS (Generally Recognized as Safe) status: see Title 21 of the Code of Federal Regulations (CFR) number 172.515. Further, BCP is the first known “dietary cannabinoid” as it interacts directly with the cannabinoid receptor type 2 (CB2) with high affinity [20]. This receptor is not psychomodulatory like its sister receptor (cannabinoid receptor type 1, CB1), but is a possible target for therapeutic intervention of inflammation and pain, to name a few [21, 27–31].

We have previously demonstrated that BCP initiates a remarkable and concerted upregulation of the majority of steps in the cholesterol biosynthetic pathway in hypoxic TNBC cells [24]. Considering the importance of oxygen status in the tumor microenvironment, here we investigate the interactive effects between BCP and hypoxia in relation to oxygen consumption, along with cholesterol and lipid homeostasis. We show that oxygen consumption is decreased in HBrC cells exposed to hypoxia relative to normoxia in keeping with the Warburg effect. Interestingly, the hypoxia-induced reduction in oxygen consumption in UFH-001 cells, which have the TNBC phenotype, was not affected by BCP, indicating that mitochondrial activity is not further altered by BCP. In addition, we show that BCP increased total cholesterol (free plus esterified) by a process that is independent of oxygen status. Yet, we discovered that the unique global lipidomic signature of hypoxia is blocked by BCP. This appears to be driven by the loss of differences in the saturation ratios of the fatty acid pools. From these data, we suggest that BCP interferes, in part, with the hypoxic signature that allows for tumor success in hypoxic environments.

## Results and discussion

### Hypoxia reduces oxygen consumption in hBrC cells which is not affected by BCP

**Fig 1:**
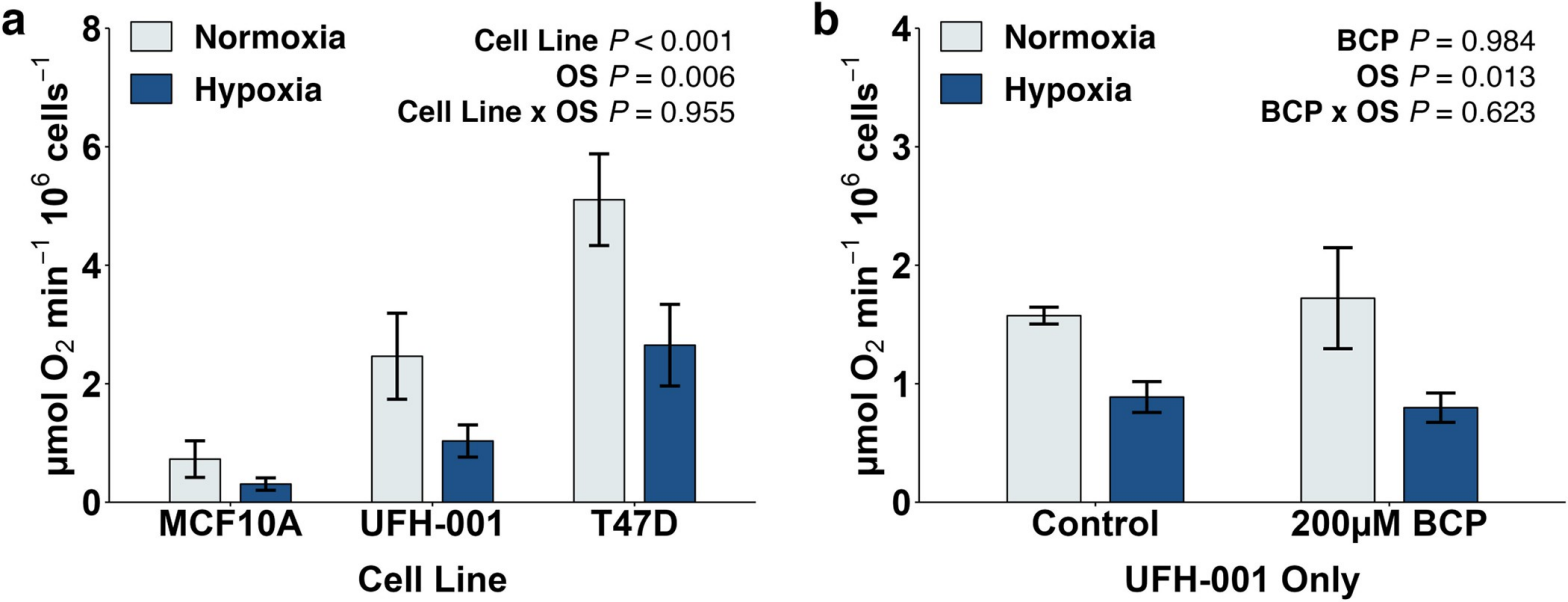
Oxygen consumption in breast cancer cells is sensitive to hypoxia but not BCP. (a) Cells were exposed to a normoxic environment or 1% oxygen in metabolic chambers. (b) In a separate experiment, UFH-001 cells were exposed to the same conditions but included 200 μM BCP. At the end of 16h, cells were washed and resuspended in 2 mL PBS containing 25mM glucose, at 37°C, and transferred to the oxygraphy to determine rates of oxygen consumption. Oxygen consumption is reported as μmol/min/10^6^ cells. Bars represent means ± S.E.M of 5 biological replicates in (a) and 3 biological replicates in (b). Data were analyzed by two-way ANOVA.

Extracellular acidification of the tumor microenvironment is a hallmark of aggressive breast cancers [3, 8]. We have previously shown that extracellular acidification, from protons arising from glycolysis, is significantly higher in both normoxic and hypoxic UFH-001 cells (triple negative phenotype) versus normoxic or hypoxic T47D cells (luminal A subtype) [32]. This was true whether extracellular pH was kept at physiological levels (pH 7.4) or at the pH found in acidic tumors (pH 6.8). This predicts that UFH-001 cells have either higher rates of glycolysis compared to T47D cells or that the UFH-001 cells are more efficient at diverting glucose carbons to lactic acid through the lactate dehydrogenase (LDH) reaction. Toward the first point, we have previously shown that total glucose consumption (as measured by loss of glucose from the medium) is not statistically different between UFH-001 and T47D cells [33] suggesting that glucose utilization is similar between the two cell types. Yet, we show here that oxygen consumption was significantly higher in both normoxic and hypoxic T47D cells compared to normoxic or hypoxic UFH-001 cells (Fig 1A). This indicates that a greater percentage of glucose carbons in T47D cells undergo total oxidation (i.e. transport into the mitochondria as pyruvate, decarboxylation via the pyruvate dehydrogenase complex, and flux through the TCA cycle coupled to oxidative phosphorylation) compared to UFH-001 cells. These two data sets were compared against the MCF10A line which serves as a control. Oxygen utilization was statistically lower in MCF10A cells than in either the UFH-001 cells or the T47D cells. This is consistent with the significantly lower glucose consumption data (for the MCF10A cells) reported earlier [33]. Note that all cells were sensitive to oxygen status (OS), as hypoxia reduced oxygen consumption across the panel of cells. The sensitivity to hypoxia is related to the stabilization of the HIF1α transcription factor which induces up (or down) regulation of a large number of genes. Previously, we have shown that MCF10A, T47D, and UFH-001 cells express HIF1α mRNA (detected by northern blotting) and HIF1α protein (detected via western blotting) under hypoxic conditions or in the presence of DFO (desferroxamine mesylate, an iron chelator which mimics the effect of hypoxia) [33]. Neither HIF1α mRNA nor protein were present in normoxic cells. Interesting, one of the genes that is upregulated by HIF1α that influence glycolytic flux is the gene that encodes for pyruvate dehydrogenase kinase [34]. In UFH-001 cells, our RNAseq data provide evidence that PDK isoform 3 is significantly upregulated (q = .0068). This enzyme phosphorylates the E1 complex of pyruvate dehydrogenase (PD) which blocks activity and thus the production of acetyl CoA [35]. This “starves” the TCA cycle relative to glucose carbons, under hypoxic conditions, which reduces oxygen consumption (Fig 1).

**Fig 2:**
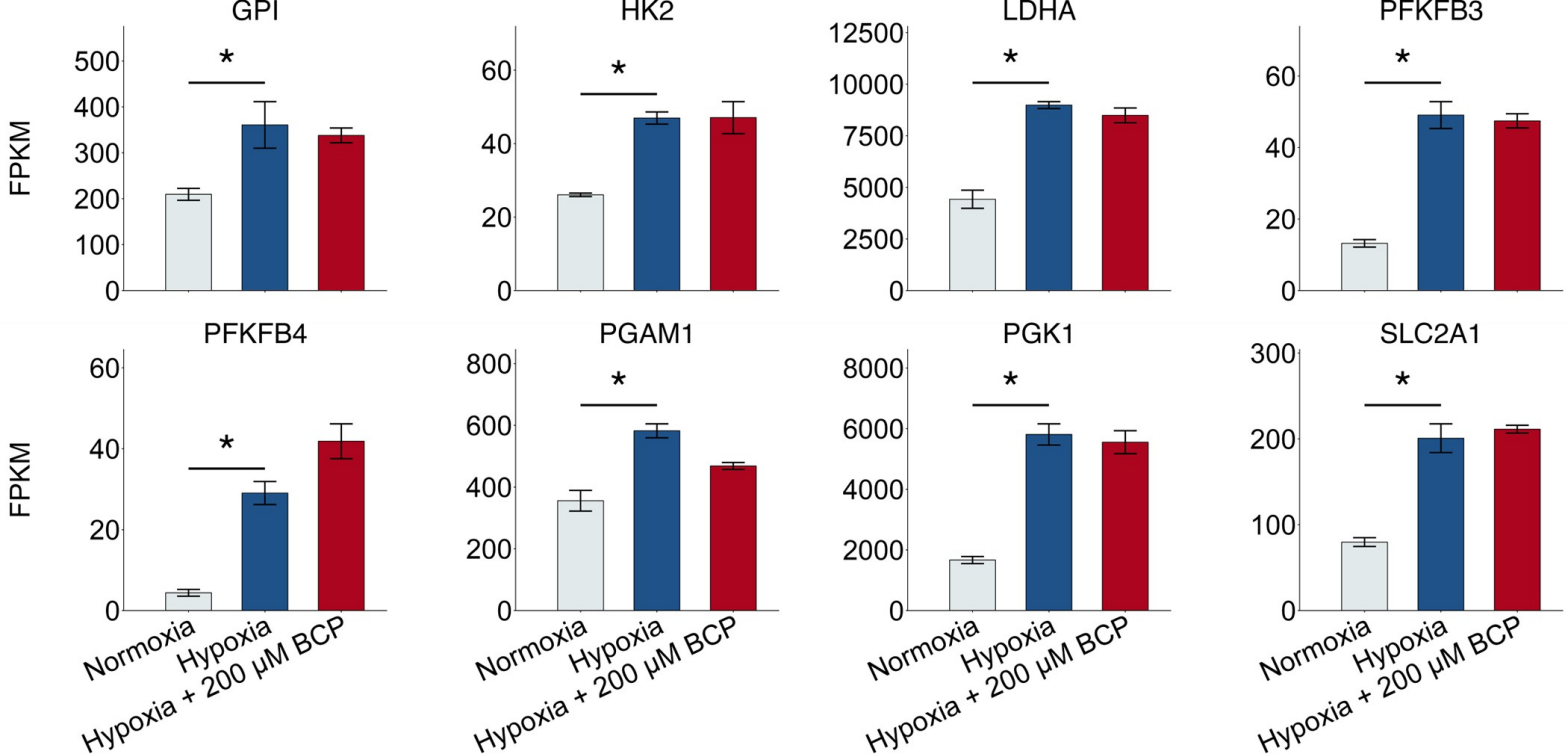
Hypoxia-sensitive glycolytic genes are not affected by BCP. RNAseq data (GSE125511), generated from cells exposed to hypoxic conditions in the presence or absence of BCP, were queried for hypoxic-sensitive glycolytic genes. Bars represent 3 independent experiments for each experimental condition with a q-value cutoff of ≤ 0.05. Asterisks connecting Normoxia and Hypoxia treatments indicate significant (q-value ≤ 0.05) effects of Hypoxia; asterisks connecting Hypoxia and BCP treatment indicate significant effects of BCP (none in this case).

With extensive gene regulation data on UFH-001 cells, gathered via RNAseq analysis, we focused on these cells to test the effect of BCP on oxygen consumption. The BCP concentration chosen for this experiment was selected because it is the highest concentration across a dose response curve that does not induce cytotoxicity but induces upregulation of the cholesterol biosynthetic pathway under hypoxic conditions [24]. Notably, BCP did not affect oxygen consumption in either normoxic or hypoxic UFH-001 cells (Fig 1B). This suggests that BCP, at the concentration used in this study, does not alter mitochondrial function. While oxygen consumption in UFH-001 cells decreased under hypoxic conditions (Fig 1A), flux through glycolysis (proton production) increased as noted above [32]. This difference is likely driven by upregulation of hypoxic-sensitive glycolytic genes in UFH-001 cells (Fig 2). Of the nine genes that encode proteins for glycolysis, four genes have q values of less than 0.05 (HK2, GPI, PGK1, and PGAM1). Three additional genes (PFKM, ALDOA, and TPI1) did not make our list, but have p values of less than 0.01, so are likely to be hypoxic sensitive. In fact, Semenza, et al., showed that PFKL, ALDOA, and PGK1 in Hep3B cells contain the HIF1 binding site in each of their promoter regions [36]. Included in our list are two genes (PFKFB3 and PFKFB4) whose translated proteins modulate the synthesis of fructose 2,6 bisphosphate which allosterically activates phosphofructokinase (PFKM). We also have included GLUT1 (SLC2A1), the plasma membrane transport protein that delivers glucose to the glycolytic pathway. Expression of this protein has been previously verified by western blotting [33]. LDH (LDHA) is also included in Fig 2. This enzyme catalyzes the conversion of pyruvate to lactate and is responsible for diverting glucose carbons away from the PD complex and thus mitochondrial oxidation. Indeed, the acidic phenotype of aggressive breast cancer tumors is in part related to the increase of lactic acid production and its release into the microenvironment [37]. BCP did not affect either the enhanced expression of glycolytic or accessory genes, the upregulation of LDH, or elevated expression of GLUT1 (Fig 2). We conclude that BCP does not alter central glucose metabolism, oxidative capacity, or viability of the UFH-001 cells. It is worth noting that hypoxic-sensitive glycolytic genes are a small subset of the 1411 genes that are altered by hypoxia. This larger set of hypoxia-induced genes, both up- and down-regulated, is shown in S1A Fig (q values ≤ 0.05) and had a strong enough effect to generate a distinct transcriptome signature relative to normoxic conditions (S1B Fig). By comparison, BCP affects transcription of a more selective gene set within the hypoxic setting (94 genes in total, q values ≤ 0.05; S1A Fig) which includes those that regulate cholesterol and lipid biosynthesis along with production of unique interleukins as previously described [24]. We have provided the complete set of differentially expressed genes in normoxic vs hypoxic and hypoxic vs hypoxia plus 200 μM BCP in S1 Table.

### BCP increases cholesterol concentration independent of oxygen status

**Fig 3:**
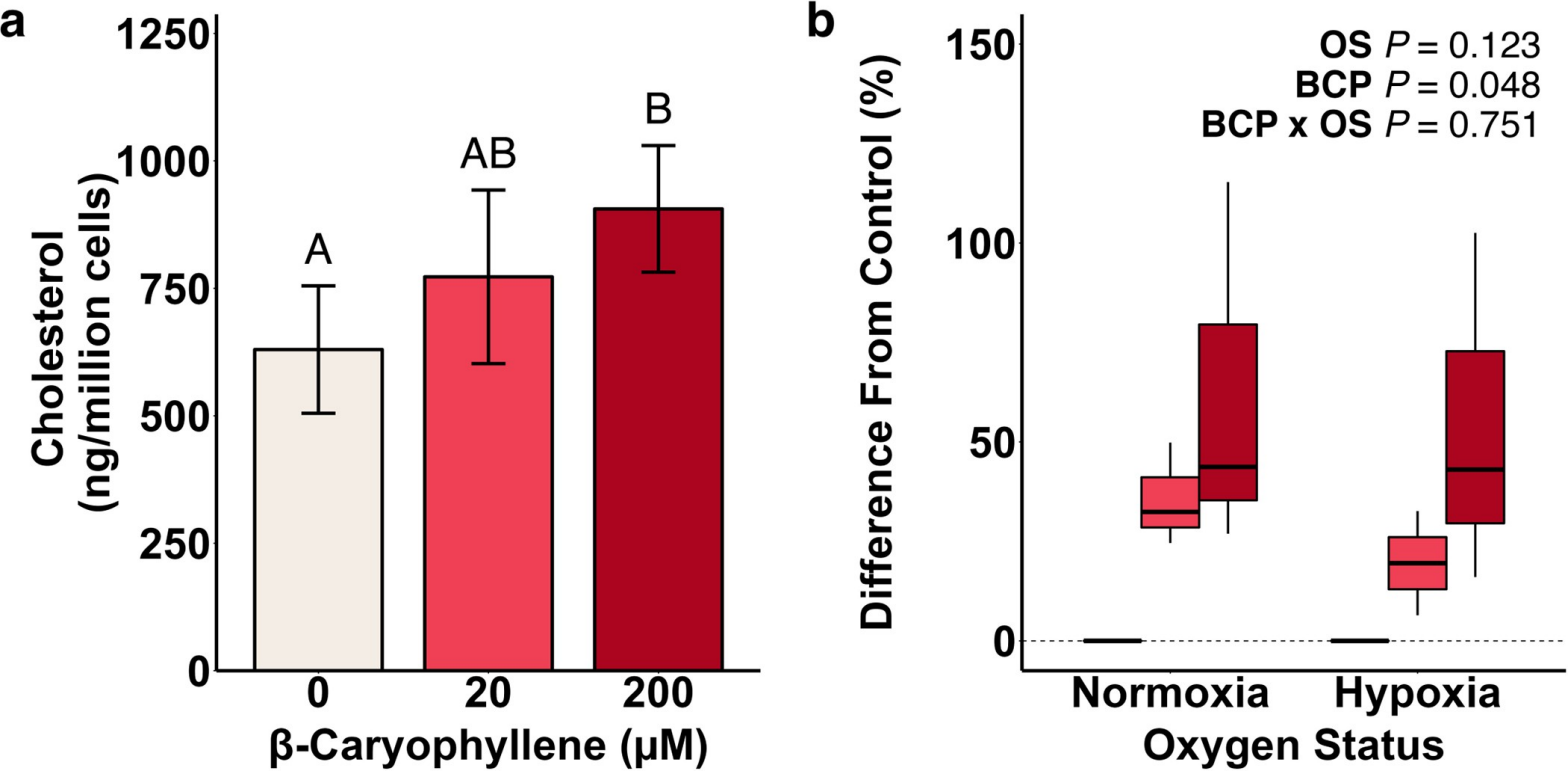
Cholesterol content of UFH-001 cells is sensitive to BCP but not hypoxia. UFH-001 cells were exposed to normoxic or hypoxic conditions in the presence of BCP for 16h. At the end of the incubation, cells were washed and processed for lipid extraction as described in Methods. (a) Total cholesterol content by BCP treatment. Bars are means ± S.E.M of 6 biological replicates. Data were log-transformed for statistical analysis; different letters above the bars indicate statistical differences at α ≤ 0.05 from the main effect of BCP in the mixed-model ANOVA. (b) Box-and-whisker plot of cholesterol content expressed as a percent difference from BCP controls. Each box represents 3 biological replicates.

Our previous transcriptional work focused on the effect of BCP in the UFH-001 cells within the hypoxic environment [24]. While this revealed a remarkable, global up-regulation of the cholesterol biosynthetic processes, it excluded the normoxic controls. In large part, this prevented conclusions regarding the specificity of BCP for the hypoxic phenotype. That said, we were able to verify that BCP increased free cholesterol content under both normoxic and hypoxic conditions at that time [24] suggesting that the increase in transcription was followed by increased translation of the involved enzymes. Yet, cholesterol esterification provides an important path for both storage and reducing free cholesterol in cells. Here, we used GC/MS to measure total cholesterol (both free and esterified) in normoxic and hypoxic UFH-001 cells. The results are nearly identical to our previous study, as indicated by the new data in Fig 3A, suggesting that the free and esterified pools are regulated to a similar extent. Cholesterol is esterified by the ER-localized enzymes acyl CoA cholesterol acyl transferase 1 (ACAT1) and acyl CoA cholesterol acyl transferase 2 (ACAT2) [38]. RNAseq data from the UFH-001 cells show that both forms are expressed at the transcriptional level. Based on transcript number, ACAT 1 is by far the predominant form. However, neither hypoxia nor BCP affected gene transcription of either of the ACAT genes. The ACAT1 gene is somewhat unique in its regulation compared to those in the central cholesterol biosynthetic pathway. There is no binding site for the SREBP family of transcription factors [39], although there are other factors that can regulate transcription through the P1 transcription site, like glucocorticoids or tumor necrosis factor [39]. Rather, the ACAT1 enzyme is activated by the binding of free cholesterol, itself [40]. So, as cholesterol increases, so does cholesterol esterification. As there was little variation in the fold difference between the effect of BCP on free [24] vs total (Fig 3A), and neither glucocorticoids nor TNF were added to our system, it is unlikely that esterification is independently modulated by factors other than cholesterol availability. We also demonstrate that the BCP-dependent increase in cholesterol in UFH-001 cells is independent of OS (Fig 3B), which confirms the strong bias in the BCP-induced changes in cholesterol biosynthetic pathways compared to the effect of hypoxia. We have also provided GO enrichment analysis to show the affected categories in hypoxia vs normoxia in S2 Table (and the genes in each category) and the pictorial representation of those data:hypoxia vs hypoxia plus BCP in S2 Fig. Moreover, we include a heat map of the DEGs identified from the original four data set experiment, run in triplicate, in S3 Fig, which underscores the relatively strong effect of hypoxia on the transcriptome-level profiles.

### Hypoxia lowers monounsaturated fatty acid species and alters unsaturated:saturated ratios

**Fig 4:**
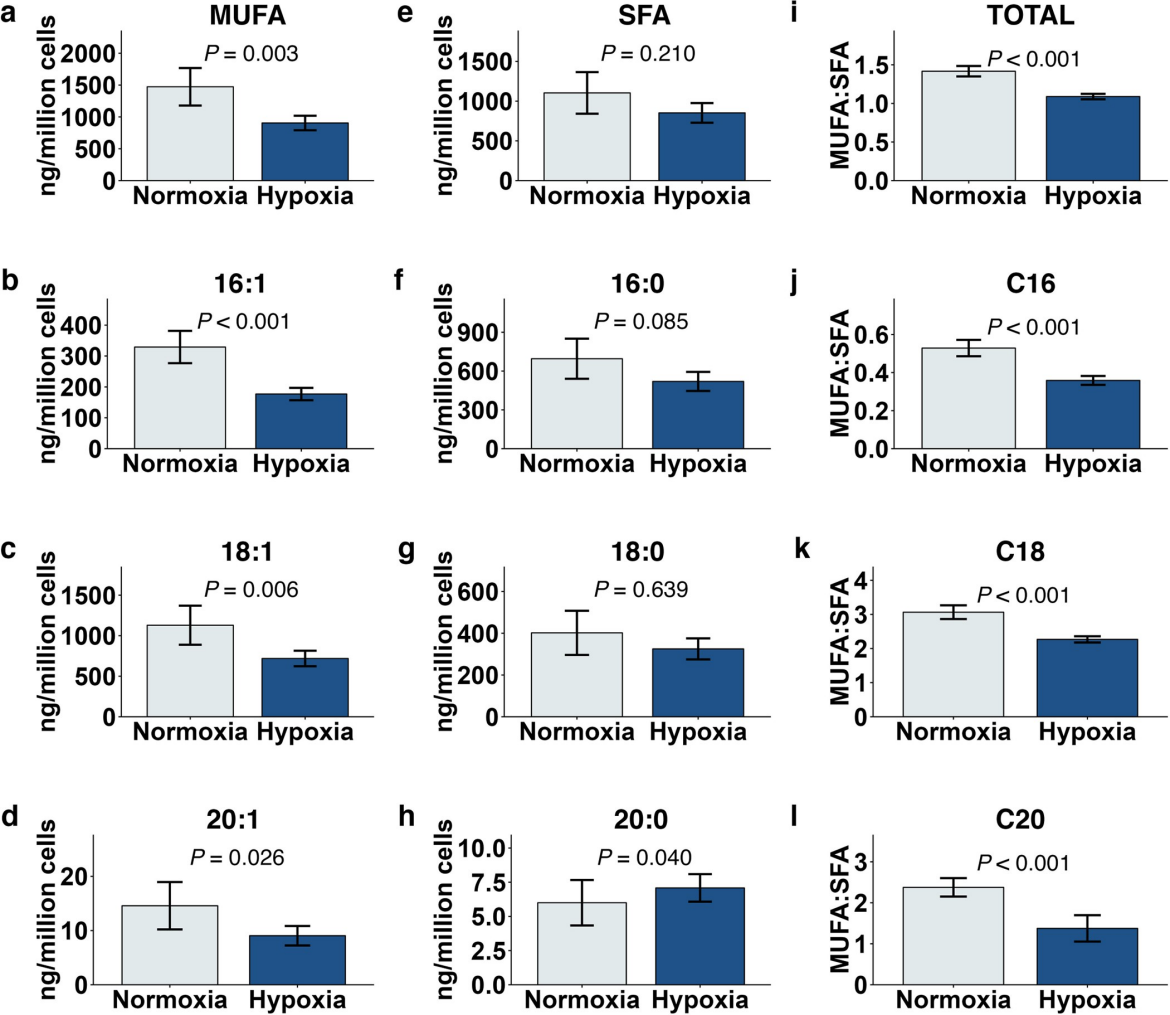
Hypoxia reduced mono-unsaturated fatty acid concentrations in UFH-001 cells independent of BCP. UFH-001 cells were exposed to normoxic or hypoxic conditions in the presence of BCP for 16h. At the end of the incubation, cells were washed and processed for lipid extraction as described in Methods. (a-d) MUFA and (e-h) SFA species as a whole or by individual acyl chain lengths; data are ng/million cells. (i-l) MUFA:SFA ratios as a whole or by individual acyl chain lengths. Bars represent means ± S.E.M of 9 biological replicates. *P*-values reported are the main effect of OS from the mixed model ANOVAs.

One of the features of cancer cells is their ability to sustain proliferative capacity [41, 42]. This requires enhanced lipid biosynthesis for membranes to support cell proliferation and to provide energy to fuel metastasis [43]. In addition, unique species of fatty acids have been identified, including higher levels of monounsaturated fatty acids (MUFA’s) over a broad range of cancer cells [44]. The thinking here is that these species, destined for phospholipids and membrane biogenesis, alter membrane dynamics and favor the construction of signaling platforms within the membrane. Indeed, it is likely that the ratio of MUFA’s to saturated fatty acids (SFA’s) and polyunsaturated fatty acids (PUFA’s) play a significant role in these processes. While the tumor microenvironment likely plays a role in lipid dynamics, the specific role of hypoxia is not well understood. In UFH-001 cells, only a few key genes in the fatty acid biosynthetic pathway were upregulated by BCP (compared to the global upregulation of cholesterol biosynthesis). This included the gene that encodes for delta-9 stearoyl CoA desaturase (SCD1). This enzyme introduces the first double bond in the cis-delta 9 position of palmitoyl CoA (C-16) and stearoyl-CoA (C-18) (and other less abundant fatty acyl CoA’s of length C12 -C19) creating monounsaturated lipids [45]. This raised the possibility that fatty acid saturation may be affected by BCP which in turn might alter fatty acid structure and lipid composition. This idea led us to consider the saturation state of lipid species and the effect that BCP and hypoxia might have on these pools. In our hands, hypoxia resulted in a comprehensive reduction in MUFA concentrations driven by values for C16:1, C18;1, and C20:1 Fas (Fig 4A–4D). In contrast, the corresponding saturated fatty acids were largely unaffected by hypoxia (Fig 4E–4G), although there was a significant increase in the very small pool of 20:0 taking into account variance between experiments (Fig 4H). Overall, the hypoxia-induced reduction in MUFA concentrations led to a decrease in the overall unsaturated:saturated FA ratios for each of the FA chain lengths (Fig 4I–4L). In light of the fact that MUFA’s are considered a hallmark of cancer, this result was unexpected. MUFA’s are preferentially used for membrane biogenesis [46] and are thought to be less susceptible to peroxidation, both of which would be advantageous for cancer cells [47]. But, not all breast cancer tissue experiences hypoxia and our experiment was designed to determine specifically the effect of hypoxia. That said, cells in 2D culture do not always behave as cells *in situ*.

### BCP mitigates the metabolomic signature of hypoxia

**Fig 5:**
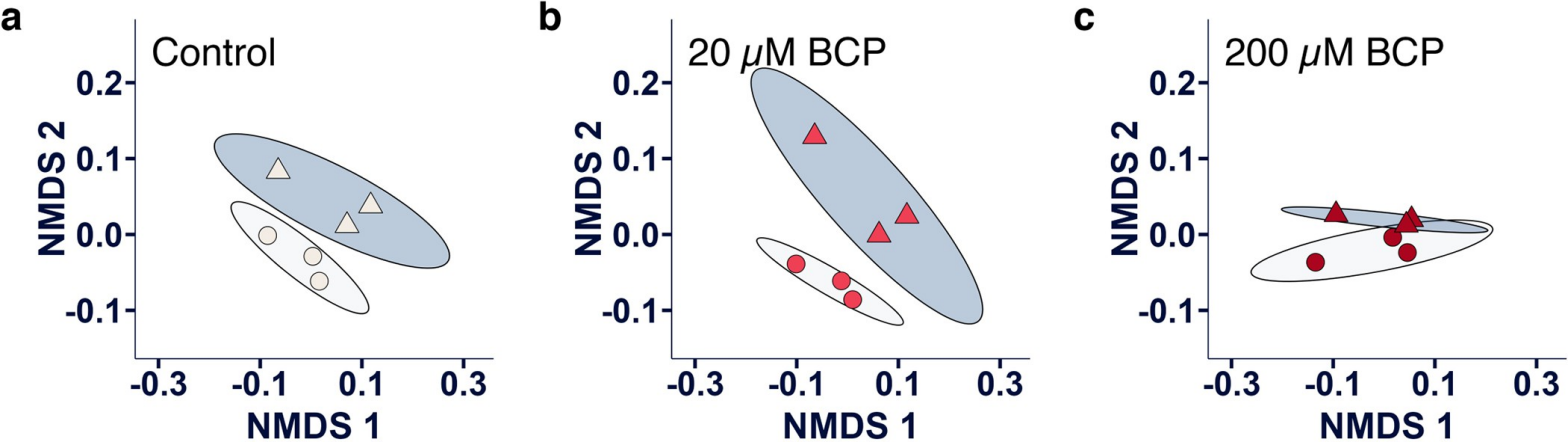
BCP attenuates the effect of the lipidomic signature of hypoxia. UFH-001 cells were exposed to normoxic or hypoxic conditions in the presence of BCP for 16h. At the end of the incubation, cells were washed and processed for lipid extraction as described in Methods. NMDS was performed on 25 analytes using MetaMDS with Bray-Curtis dissimilarities. The distances between points reflects their relative similarity to or difference from each other. Circular points represent cells in normoxic conditions; triangular points represent cells in hypoxic conditions. Light-shaded ellipse regions are the 95% confidence areas for the normoxic samples; darker-shaded ellipse regions are the 95% confidence areas for the hypoxic samples. (a) Control conditions without BCP, (b) 20 μM BCP, (c) 200 μM BCP.

**Fig 6:**
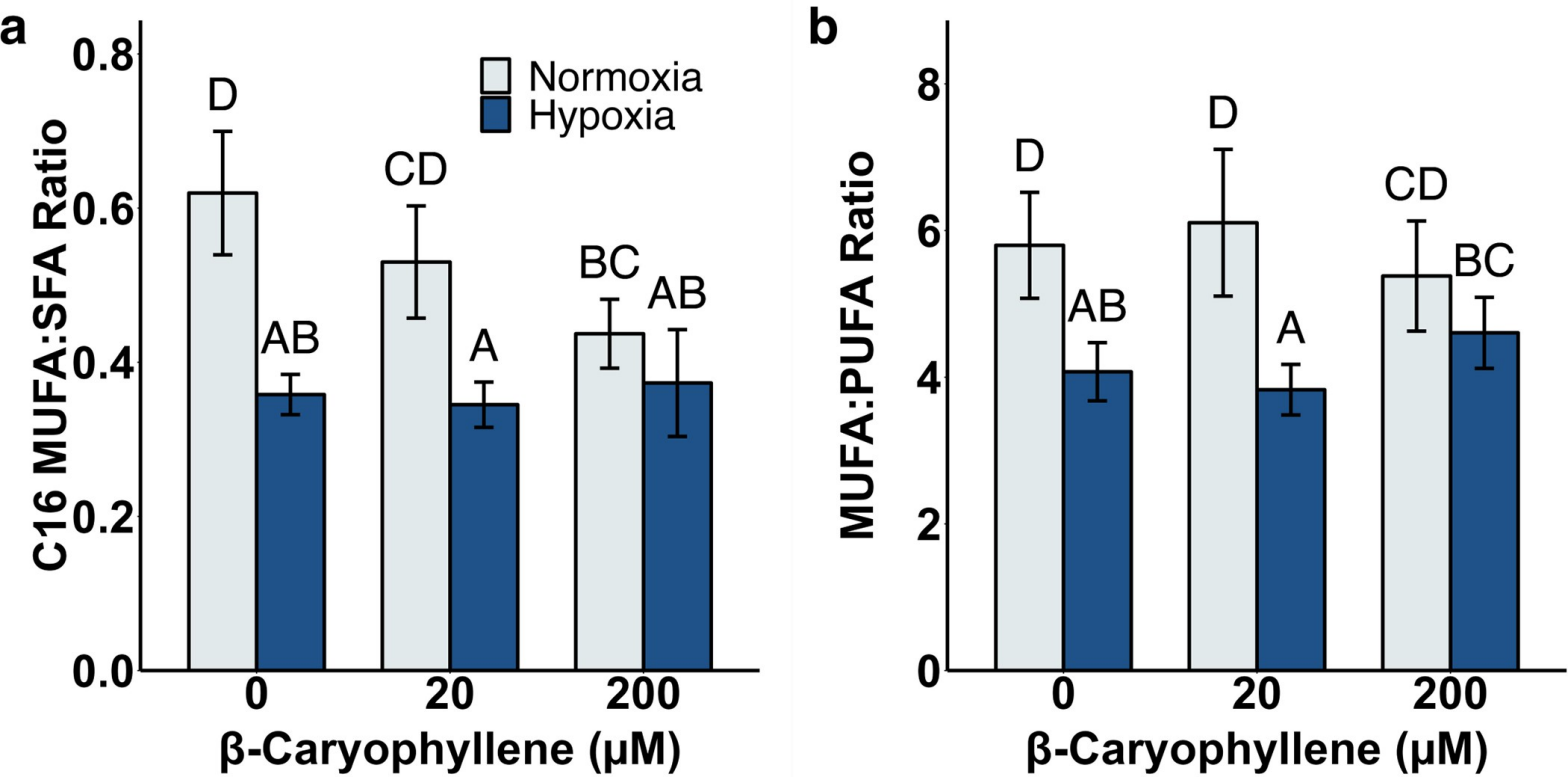
BCP attenuates the effect of hypoxia on fatty acyl saturation ratios. UFH-001 cells were exposed to normoxic or hypoxic conditions in the presence of BCP for 16h. At the end of the incubation, cells were washed and processed for lipid extraction as described in Methods. Bars are means ± S.E.M. of 3 biological replicates. Data were log-transformed for statistical analysis; different letters above the bars indicate statistical differences at α ≤ 0.05. (a) C16 MUFA:SFA [palmitoleic:palmitic] ratio by hypoxia and BCP treatments. (b) MUFA:PUFA ratio by hypoxia and BCP treatments.

Nonmetric multidimensional scaling (NMDS) is an ordination that is used to visualize complex multivariate datasets in a reduced number of dimensions based on dissimilarity matrices. Here, NMDS was used to consolidate all unique lipid analytes to generate a “lipidomic signature” for each treatment group (Fig 5). Based on 95% confidence areas, hypoxia produced a distinct lipidomic signature in the absence of BCP (Fig 5A) that was maintained in the presence of 20 μM BCP (Fig 5B) but not in the presence of 200 μM BCP (Fig 5C). This suggests that 200 μM BCP interferes with the hypoxic phenotype. The drivers of the 200 μM BCP signature may reside in the relative pool sizes of the fatty acids. In particular, hypoxia alone reduced the ratio of 16:1 to 16:0, but this effect was eliminated in the presence of 200 μM BCP (Fig 6A). Moreover, a similar BCP-dependent effect was also observed between ratios of total pools of MUFAs to polyunsaturated fatty acids (PUFAs) (Fig 6B).

### Transcriptional signatures of stress are affected by hypoxia

**Fig 7:**
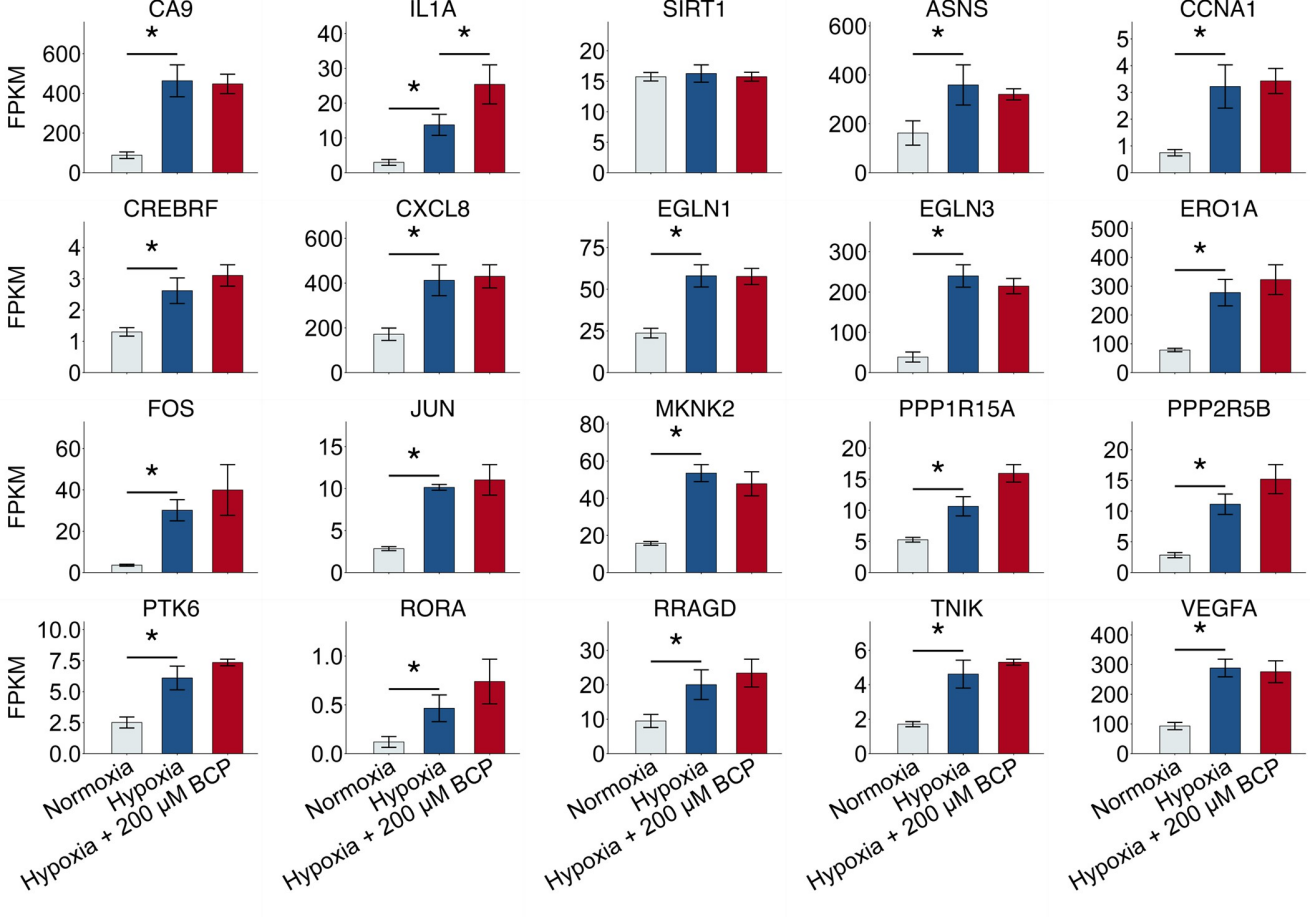
Hypoxia-sensitive stress genes are largely unaffected by BCP. RNAseq data (GSE125511) generated from cells exposed to hypoxic conditions in the presence or absence of BCP were queried for hypoxic-sensitive glycolytic genes. Bars represent 3 independent experiments for each experimental condition with a q-value cutoff of ≤ 0.05. Asterisks connecting Normoxia and Hypoxia treatments indicate significant (q-value ≤ 0.05) effects of Hypoxia; asterisks connecting Hypoxia and BCP treatment indicate significant effects of BCP (Interleukin1, alpha subunit [IL1A] only).

Because oxygen deprivation is in itself a stress, we also examined the set of “stress” genes, identified through and by Reactome.com from RNAseq analysis in normoxic vs hypoxic UFH-001 cells (Δ log 1, q values < .05) (Fig 7). Interestingly, this set of 20 genes includes CA9, the gene that encodes for carbonic anhydrase IX (CAIX). We have shown that CAIX mRNA, protein, and activity is increased in UFH-001 cells by exposure to hypoxia [33, 48] to a similar extent as is CA9 gene upregulation (~ 5-fold). CAIX is a biomarker for hypoxic regions of breast tumors [49], is associated with ER-negative breast tumors [50, 51], and is an indicator of poor prognosis in breast cancer patients [50, 52, 53]. We have also shown that CAIX is a major contributor to pH control in triple negative breast cancer cells, like UFH-001 cells [32, 54]. While CA9 and 18 of the other stress genes are influenced by OS, only one of the 20 stress genes is further affected by BCP, i.e., IL1A, interleukin-1α. We noted this BCP effect previously, including the enrichment of both IL1 and IL10 signaling [24]. Overall, IL-1α stimulates the inflammatory response inducing the release of prostaglandins (which are generated from the long chain fatty acid pools). IL-1α also regulates transcription in response to a wide range of stimuli (almost always with TNFα), can activate signaling from its cognate receptor (IL-1R1) at the plasma membrane, or serve as an endogenous alarmin [55]. These features suggest that IL-1α acts to gauge the magnitude of incoming stress or damage and then initiate an inflammatory process or steer a reparative process.

### CB_2_ receptor signaling does not likely underlie the BCP-induced effects on lipid composition

**Fig 8:**
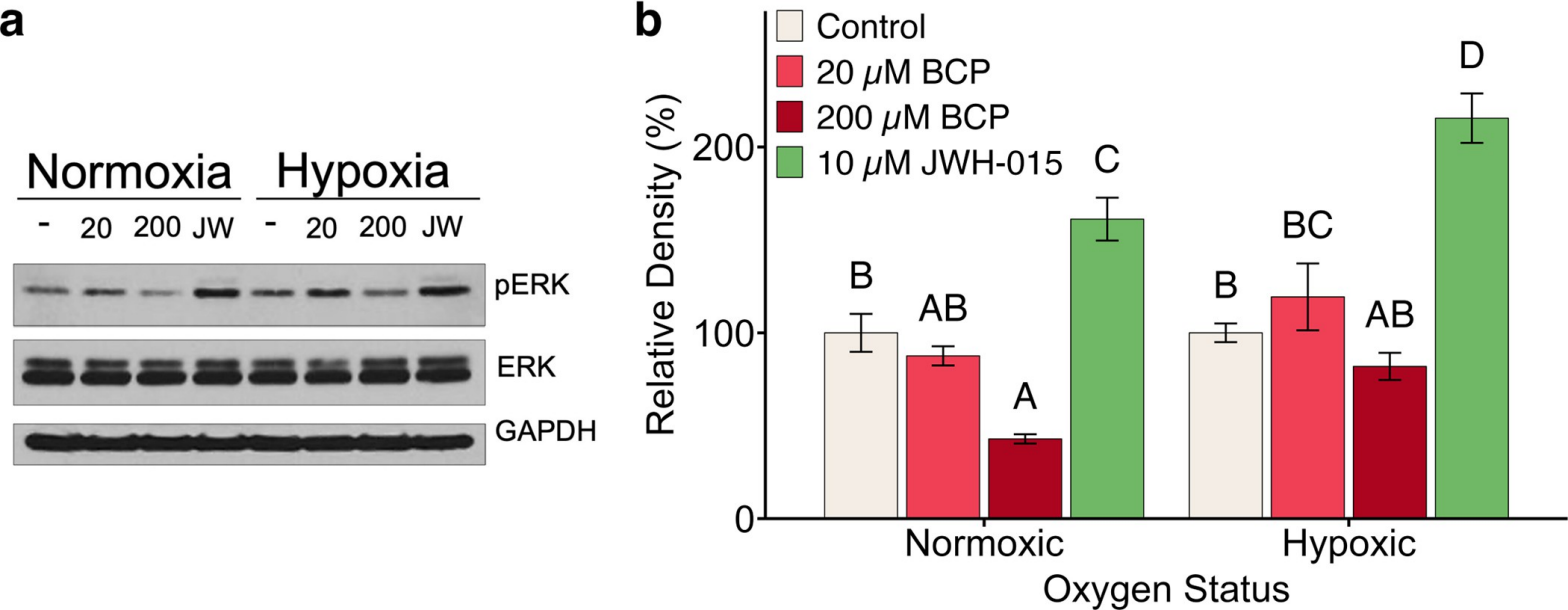
pERK expression is reduced in the presence of 200 μM BCP. Panel a. UFH-001 cells were exposed to normoxic or hypoxic conditions for 16h in the presence or absence of BCP. A second set of UFH-001 cells was exposed to normoxic or hypoxic conditions for 15 ½ h after which JWH-015 (10μM) was added for the final 30 min (total of 16h of normoxia and hypoxia). Cells were washed with PBS and lysed with RIPA buffer containing protease inhibitors. Equal protein concentrations from the soluble phase were used to load SDS-PAGE gels which were then transferred to nitrocellulose for analysis of ERK, pERK and GAPDH (loading control). Panel b. These data represent the relative densities of the bands in Panel a where the bars represent the means ± S.E.M of 3–5 replicates. Data were analyzed by two-way ANOVA.

BCP is able to interact with the CB_2_ receptor with nM affinity although the concentrations that affect signaling are in the low μM range [20]. The CB_2_ receptor is a member of the G-protein coupled receptors. Activated CB_2_ receptors inhibit adenylate cyclase through their GiGo-α subunits [56, 57]. At the same time, the βγ complex is released which interacts with and activates MAP kinase, specifically the extracellular signal-regulating kinases (ERK1/2) [56–58]. Here, we have examined the potential for BCP to activate ERK1/2 (Fig 8). In this experiment, we have compared the chronic treatment of UFH-001 cells with BCP (the condition that leads to activation of the cholesterol biosynthetic path) with the acute activation of the CB_2_ receptor agonist, JWH-015. In our hands, JWH-015 significantly increased the phosphorylation of ERK1/2, moreso in hypoxic than normoxic cells. In normoxic cells, BCP had no effect at 20 μM but reduced phosphorylation of ERK1/2 at 200 μM. In hypoxic cells, neither concentration of BCP affected ERK1/2 phosphorylation. These data suggest that the mechanism by which BCP affects cholesterol and lipid homeostasis, in our system, may be independent of CB_2_ receptor activation. However, these data differ from that presented by Hanlon et al. who showed that JHW-015, at the same concentration used in our study, reduced ERK phosphorylation [59]. In fact, they suggested that JHW-015 action was also independent of CB_2_ activation. The difference between our studies is not readily apparent, although they did use 4T1 cells, a mouse breast cancer line. Other investigators have identified mechanisms that, aside from CB_2_ activation, may underlie the anti-proliferative effects of BCP. For example, BCP increases phosphatidylserine exposure at the plasma membrane which serves as an apoptotic signal [60]. This suggests that the plasma membrane is a BCP target, which is consistent with our study. Alternatively, BCP reduces proliferation through miR-659-3p which reduces the expression of sphingosine kinase I [61], the gene for which (SPHK1) has many features of an oncogene and is highly expressed in cancers, including breast cancer. BCP also reduces proliferation by blocking the STAT3/mTOR/AKT signaling path [62]. However, our RNAseq data from UFH-001 cells show that neither hypoxia nor BCP treatment affects either SPHK1 or STAT3. These observations do not necessarily exclude the mechanistic involvement of these pathways for BCP action on cholesterol or lipid homeostasis that we have identified, but rather indicate that further time course and concentration studies are critical to determine their role.

## Conclusions

Over the last decade, numerous studies have shown that BCP exhibits anti-proliferative properties across various carcinoma cell lines [23, 60–66] but hypoxia was not addressed as a specific issue despite the hypoxic nature of most solid tumors [67]. In the present study, we hypothesized that BCP might reverse the hypoxic phenotype of TNBC cells. While hypoxia affected a number of metabolic indices including the enhanced transcription of glycolytic and stress genes, flux through glycolysis, and reduced oxygen consumption, BCP by-in-large did not affect these parameters. We showed that BCP increases total cholesterol content and that this effect was independent of oxygen status. The most convincing evidence that BCP interferes with the hypoxic phenotype in our study is the loss of the unique lipodomic signature in hypoxic cells treated with the highest concentration of BCP, that we previously showed enhanced cholesterol biosynthesis without inducing cytotoxicity [24]. This signature was comprised of 32 unique analytes but was predominated by C16, C18, and C20 fatty acids. Hypoxia consistently reduced the concentration of MUFAs and thus reduced the ratio of MUFAs to SFAs. Each concentration of BCP reversed this difference in a stepwise manner. In addition, the hypoxia-driven decrease in the ratio of MUFAs to PUFAs was not apparent in the presence the highest concentration of BCP. Ultimately, our results suggest that BCP may interfere with the lipid signature modulated by hypoxia which could have consequences for membrane biosynthesis or composition, both of which are important for cell growth.

## Materials and methods

### Cell culture

All cell lines were authenticated. MCF10A cells were a gift from Dr. Brian Law. These cells are used as a control for breast cancer cells in culture. These cells were isolated from women with fibrocystic disease which became immortalized in culture [68]. While these cells are not transformed, they do have a progenitor (basal-like) phenotype [69]. Dr. Keith Robertson provided T47D cells. These cells were derived from pleural effusion of a patient with breast carcinoma [70] and have a luminal A phenotype and express the estrogen receptor (ER+) but not the HER2 receptor [69]. The UFH-001 line was developed and characterized in the lab of Dr. Susan Frost [71, 72]. This line has a triple negative phenotype, are fast growing, form spheroid structures in culture, and generate tumors in an immunocompromised mouse model. Each line was maintained at 37°C, at 5% CO_2_. MCF10A cells were cultivated in Dulbecco’s Modified Eagle’s Medium (DMEM/Ham’s F12 medium (1:1)) (Corning Lellgro) supplemented with 5% horse serum (Sigma Aldrich), 10 μg/mL insulin (Eli Lilly), 20 ng/mL epidermal growth factor (EGF) (Upstate Biochem), and 100 ng/mL dexamethasone (Sigma Aldrich). The T47D cells were maintained in McCoy’s medium (Gibco) containing 10% fetal bovine serum (FBS; Sigma Aldrich) and 1μg/mL bovine insulin (Elanco). While these cells are ER+, they do not require the addition of estrogen for growth. The UFH-001 cell lines were cultivated in DMEM supplemented with 10% FBS.

### Oxygen consumption

Oxygen consumption was measured using a HansaTech Oxygraph+ System. Briefly, T47D, MCF10A, and UFH-001 cells were grown in 10 cm dishes and were exposed to normoxic or hypoxic conditions (the latter of which were placed in Billups-Rothenberg Metabolic Chambers and exposed to 1% O_2_, 5% CO_2_, and balanced N_2_) at 37°C for 16h. In separate experiments, UFH-001 cells were exposed to 200 μM BCP for 16 h under normoxic and hypoxic conditions. The controls for the BCP-treated cells included DMSO, which was used as the solvent for the BCP. Each plate was washed twice with 5 mL ice cold PBS. A third wash was done with 1 mL of warm PBS with 25 mM glucose. Cells were gently scraped from the plate, transferred to a polystyrene culture test tube, and collected by centrifugation for 5 min at 1000 rpm at room temperature. The supernatant was then removed, and the cell pellet was gently resuspended in 2 mL PBS at 37°C containing 25 mM glucose. Cell number was determined using a Coulter counter. Oxygen consumption rates are reported as μmol/min/10^6^ cells. Three biological replicates were performed for each cell type and condition.

### RNA isolation and RNAseq

To measure the effect of hypoxia on gene expression patterns, we used RNAseq technology as part of a larger project that has been deposited in NCBI’s Gene Expression Omnibus (29) and are accessible through GEO Series accession number GSE125511 (https://www.ncbi.nih.gov/geo/query/acc.cgi?acc=GSE125511). Briefly, RNA (RIN >9) was extracted from UFH-001 cells, previously exposed to normoxia or hypoxia for 16h in the presence or absence of BCP at either 20 μM or 200 μM (RNase easy plus mini kit from Qiagen). Libraries were prepared at the Genomics Core at the University of Louisville and sequencing was performed on triplicate biological replicates (Illunima NestSeq 500), generating over 144 million 75 bp reads that aligned to the human genome (96.3% alignment rate), or approximately 24 million reads per sample. The RNAseq data were analyzed using the tuxedo suite pipeline (fastqc, trimmomatic, tophat2, cufflinks, and cuffnorm) by the KBRIN Bioinformatics Core. Differential gene expression between hypoxia and normoxia was determined with cuffdiff with a q-value cutoff ≤ 0.05.

### Total cholesterol and fatty acid quantification

UFH-001 cells (in 100 mm plates) were exposed to normoxic or hypoxic conditions (as described above) in the presence or absence of BCP (20 or 200 μM) for the times indicated. Cells were washed (3X) with ice cold sterile saline (.0.9% NaCl). The final wash was removed, and methanol (5 mL) was added to the cells and scraped into 25 mL separating flasks. Ten mL of chloroform was added to each flask, shaken, and then left at room temperature for 30 min. Five mL of ice-cold ddH_2_O was added and mixed. This was placed in a cold room for 72 h to separate the phases. The chloroform phase was collected and dried under nitrogen. Dried samples were reconstituted in chloroform and a subsample was evaporated to dryness under N_2_. Phospholipids were hydrolyzed with 250 μL 1M KOH in methanol at 50°C for 3 h, followed by 250 μL 6M HCl in MeOH for 15 min at 80°C. FAMEs and sterols were extracted with Diethyl Ether: Hexane (1:1). After evaporating to dryness, silylation of the sterols occurred with 50 μl BSTFA:TCMS (99:1) and 10 mL anhydrous pyridine for 30 min at 37°C. One mL of this solution was injected into an Agilent 7890B Gas Chromatogram in splitless mode with an inlet temperature of 300°C and a DB-5 analytical column (30 m length, 0.25 mm diameter, with a built-in 10 m DuraGuard pre-column) with a flow of 1.12 mL/min (average velocity 23.5 cm/sec). Thermal ramping initiated at 80°C for 1 min, and then ramped 20°C/min to 200°C and then 10°C/min to 325°C and held for 10 min. Analytes were detected with an Agilent 5977A Mass Spectrometer with an EI ion source with the MS in scanning mode (50–600 m/z) and transfer line and ion source temperatures set at 230°C and 150°C, respectively. Peaks within a sample were deconvoluted using MassHunter (Agilent) software and preliminary analyte identities were assigned based on comparison to the NIST14 library [73] and in-house mass spectral libraries [74]. Spectral match factor thresholds for identified metabolites was > 85%. Individual metabolites were aligned across samples using an in-house R script implemented in R (4.0.3) through RStudio (2021.09.2 Build 382) using a combination of nearest neighbor and mass spectral similarity indices.

### Lysate preparation and western blot analysis

UFH-001 cells were exposed to normoxic or hypoxic conditions with or without BCP, at the indicated concentrations, for a total of 16 h. A second set of cells were first exposed to normoxic or hypoxic conditions for 15 ½ h, and then treated for an additional 30 min to JWH-015 at 10 μM (a CB_2_ receptor activator). All cells were then placed on ice, washed with ice-cold PBS (10 mM sodium phosphate salts, 120 mM NaCl, pH 7.4), and lysed in RIPA buffer [1% NP-40, 10 mM phosphate buffer, 0.1% SDS, 150 mM NaCl, 0.5% sodium deoxycholate, 1 mM sodium orthovanadate, 0.5 mM phenylmethyl sulfonyl fluoride (PMSF) and protease inhibitor (Roche Diagnostics), pH, 7.4]. Cell lysates were clarified by centrifugation at 16,000 × g for 15 min at 4°C. Protein concentration of the clarified supernatants was determined using a modification of the Lowry assay [75]. Equal protein was loaded onto 10% PAGE gels, separated by electrophoresis according to Laemmli *et al*. [76], and transferred to nitrocellulose membranes for western blot analysis [77] using enhanced chemiluminescence (ECL) (GE Healthcare, # RPN2106 or RPN2232). Protein loading was checked by blotting for GAPDH (Cell Signaling, D16H11). Membranes were further probed for total ERK (Calbiochem #442700) or pERK1/2 (Biolabs #9106). Images of the original western blots can be found in the Supplemental material (S1 Raw images).

### Statistical evaluation

Data were analyzed in R (4.0.3) implemented in RStudio (2021.09.2 Build 382). Metabolite concentrations were analyzed with linear mixed models (lmer in the lme4 package) with oxygen status (2 levels) and BCP (3 levels) as fixed effects and Experiment as a random effect. Analyte data were log-transformed for analysis. Statistical differences were assessed by ANOVA (Anova in the car package) using Type II Wald F tests with Kenward-Roger degrees of freedom. Differences among the three BCP groups was determined by Tukey HSD tests (glht in the *multcomp* package). Oxygen consumption data were analyzed by two-way ANOVA with Tukey HSD tests for significance among groups.

Nonmetric multidimensional scale (NMDS) analysis is a powerful tool to investigate relational patterns in transcriptome and lipidome profiles. NMDS allows the representation of high-dimensional data in low-dimensional space based while maintaining the similarities between data points, which has been useful for consolidating both transcriptomic and metabolomic data. NMDS was used here to assess global patterns of hypoxia and BCP treatments on UFH-001 cells. NMDS was performed on standardized lipodomic (25 analytes) and transcriptomic (20,209 gene rows) data using Bray-Curtis dissimilaries with the metaMDS function in the vegan package in R (35). The number of output dimensions was constrained to 2. The maximum number of random starts was set to 50 and the lipid and gene expression solutions were each achieved in 20 iterations. The ordellipse function (vegan) was used to generate 95% confidence ranges in the 2-dimensional NMDS plotting space wherein non-overlapping ellipses indicate statistical separation at α = 0.05.

The statistical outputs for all data (organized by Figure number) are presented in Excel as a Supplemental file (S1 Table).

## Supporting information

S1 Fig Differential gene and transcriptome analysis. Panel a. Number of differentially expressed genes (q-value < 0.05 and log2 FC < 0) for two pairwise comparisons: Normoxic v. Hypoxic and Hypoxic v. Hypoxic + 200 μM BCP. Red and blue bars indicate genes with higher and lower expression, respectively, in the "treatment" group (e.g., "Hypoxia" in comparison 1 and "Hypoxia + 200 μM BCP" in comparison 2). Panel b Nonmetric MultiDimensional Scaling (NMDS) transcriptome analysis. NMDS was performed on a list of 20209 (of the 60603) Ensemble Gene ID that had sum and median FPKM values > 0 (i.e., were expressed in the cells) using Bray-Curtis disimilarities with a maximum dimension of 2 and a maximum of 50 iterations. 95% confidence ellipses were generated with vegan::ordellipse. Light-filled triangles represent Normoxia replicates. Light-filled diamonds represent Hypoxia replicates. Red squares represent Hypoxia + 20 μM BCP replicates. Dark red circles represent Hypoxia + 200 μM BCP replicates. Light-shaded ellipse is the 95% CI for Normoxia samples. Blue-shaded ellipses are 95% CI for each of the three Hypoxia treatment groups. Numerical outputs for NMDS coordinates, 95% confidence ellipse coordinates, and treatment centroids are in S1 Table.
(TIF)

S2 Fig Enriched GO:BP results from category compare. Nodes represent enriched annotations for DEGs. Edges represent relationship between annotations sharing high number of genes with pvalue cutoff 0.001 and edge weight greater than 0.90. Gene ontology (GO) enrichment analysis was performed using topGO with Fisher’s exact test. GO enrichment analysis was performed by the KBRIN Bioinformatics Core.
(PNG)

S3 Fig Differential expression heat map. Genes showing a differential expression |Log2FC| ≥0 in at least one of the four pairwise comparisons (Normoxic v. Hypoxic, Hypoxic v. Hypoxic + 20 μM BCP, Hypoxic v. Hypoxic + 200 μM BCP, Hypoxic + 20 μM BCP v. Hypoxic + 200 μM BCP). Individual samples are clustered from left to right—Normoxic, Hypoxic + 20 μM BCP, Hypoxic, Hypoxic + 200 μM BCP. Heatmap analysis was performed by the KBRIN Bioinformatics Core.
(PNG)

S1 Raw images PDF version of raw original western blot images for GAPDH, ERK, and pERK expression that are presented in Fig 8A. Individual films for each of the proteins assessed.
(PDF)

S1 Table Data sets for all Figs 1–8 and S1 Fig. File contains the supplemental tables described in the manuscript.
(XLSX)

S2 Table GO categories from S2 Fig. File contains supplemental tablular outputs for the GO category analysis.
(XLSX)

## Abbreviations

BCP: β-caryophyllene
TNBC: triple negative breast cancer
SCD: stearoyl-CoA desaturase
HBrC: human breast cancer cells
LDH: lactate dehydrogenase
ER: endoplasmic reticulum
HMGCR: HMG CoA reductase
ERK: extracellular signal-regulated kinase. MAPK, mitogen activated protein kinase
HIF: hypoxia-inducible factor
PDK: pyruvate dehydrogenase kinase
PD: pyruvate dehydrogenase (complex)
HK2: hexokinase, isoform 2
GPI: glucose phosphate isomerase
PGK1: phosphoglycerate kinase, isoform 1
PGAM1: phosphoglycerate mutase, isoform 1
PFKM: phosphofructokinase, muscle isoform
PFKL: phosphofructokinase, liver isoform
TPI1: triose phosphate isomerase, isoform 1
PFKFB3: 6-phosphofructo-2-kinase/fructose 2,6 bisphosphatase, isoform 3
PFKFB4: 6-phosphofructo-2-kinase/fructose 2,6 bisphosphatase, isoform 4
GLUT1: glucose transport, isoform 1
LDHA: lactate dehydrogenase, isoform A
MUFAs: monounsaturated fatty acids
SAFs: saturated fatty acids
PUFAs: polyunsaturated fatty acids
SCD1: stearoyl CoA desaturase, isoform 1
SPHK1: sphingosine kinase isoform 1
STAT3: signal transducer and activator of transcription isoform 3
NMDS: nonmetric multidimensional scaling
