# Supplementary figures and images for: Effects of β-caryophyllene and oxygen availability on cholesterol and fatty acids in breast cancer cells
Source: PLoS One. 2023 Mar 9;18(3):e0281396. doi: 10.1371/journal.pone.0281396 (PMC9997903; doi:10.1371/journal.pone.0281396)

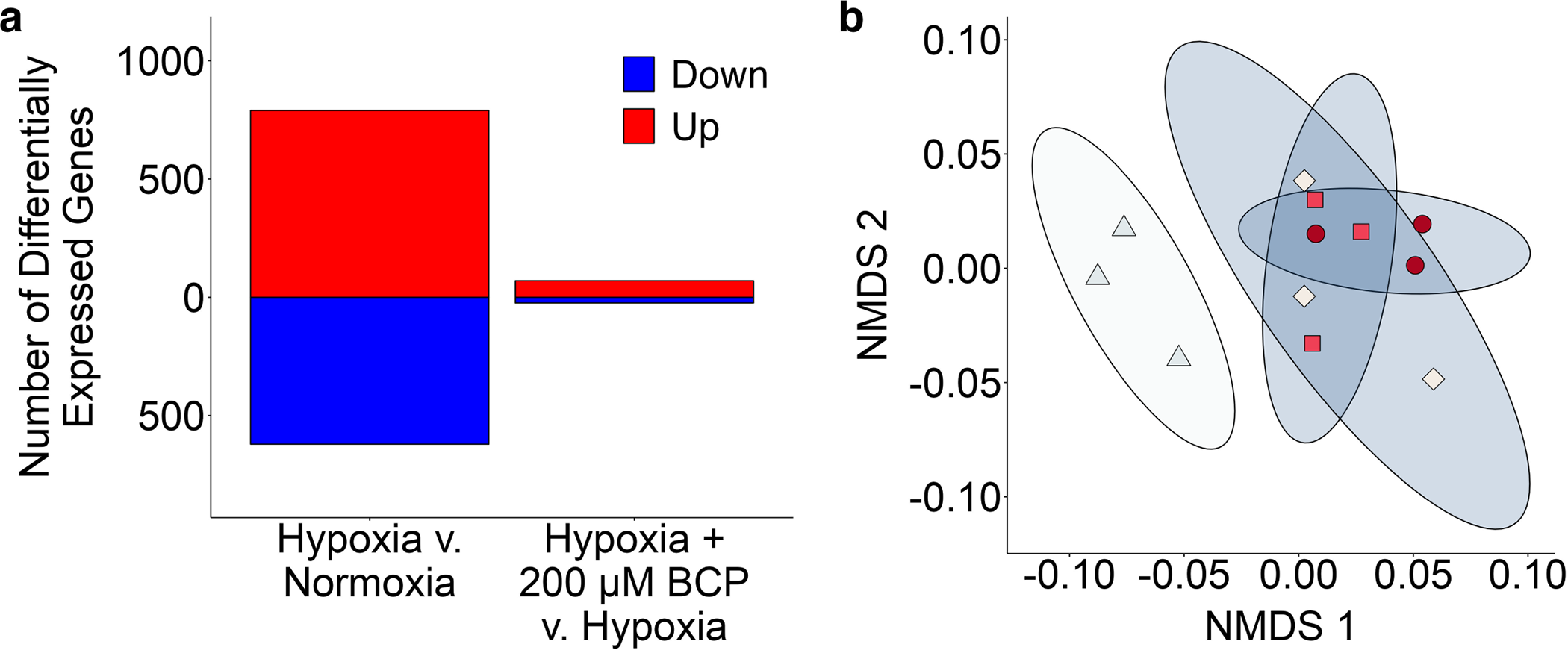

Supplement: S1 Fig — Panel a. Number of differentially expressed genes (q-value < 0.05 and log2 FC < 0) for two pairwise comparisons: Normoxic v. Hypoxic and Hypoxic v. Hypoxic + 200 μM BCP. Red and blue bars indicate genes with higher and lower expression, respectively, in the "treatment" group (e.g., "Hypoxia" in comparison 1 and "Hypoxia + 200 μM BCP" in comparison 2). Panel b Nonmetric MultiDimensional Scaling (NMDS) transcriptome analysis. NMDS was performed on a list of 20209 (of the 60603) Ensemble Gene ID that had sum and median FPKM values > 0 (i.e., were expressed in the cells) using Bray-Curtis disimilarities with a maximum dimension of 2 and a maximum of 50 iterations. 95% confidence ellipses were generated with vegan::ordellipse. Light-filled triangles represent Normoxia replicates. Light-filled diamonds represent Hypoxia replicates. Red squares represent Hypoxia + 20 μM BCP replicates. Dark red circles represent Hypoxia + 200 μM BCP replicates. Light-shaded ellipse is the 95% CI for Normoxia samples. Blue-shaded ellipses are 95% CI for each of the three Hypoxia treatment groups. Numerical outputs for NMDS coordinates, 95% confidence ellipse coordinates, and treatment centroids are in S1 Table. (TIF) [file pone.0281396.s001.tif]

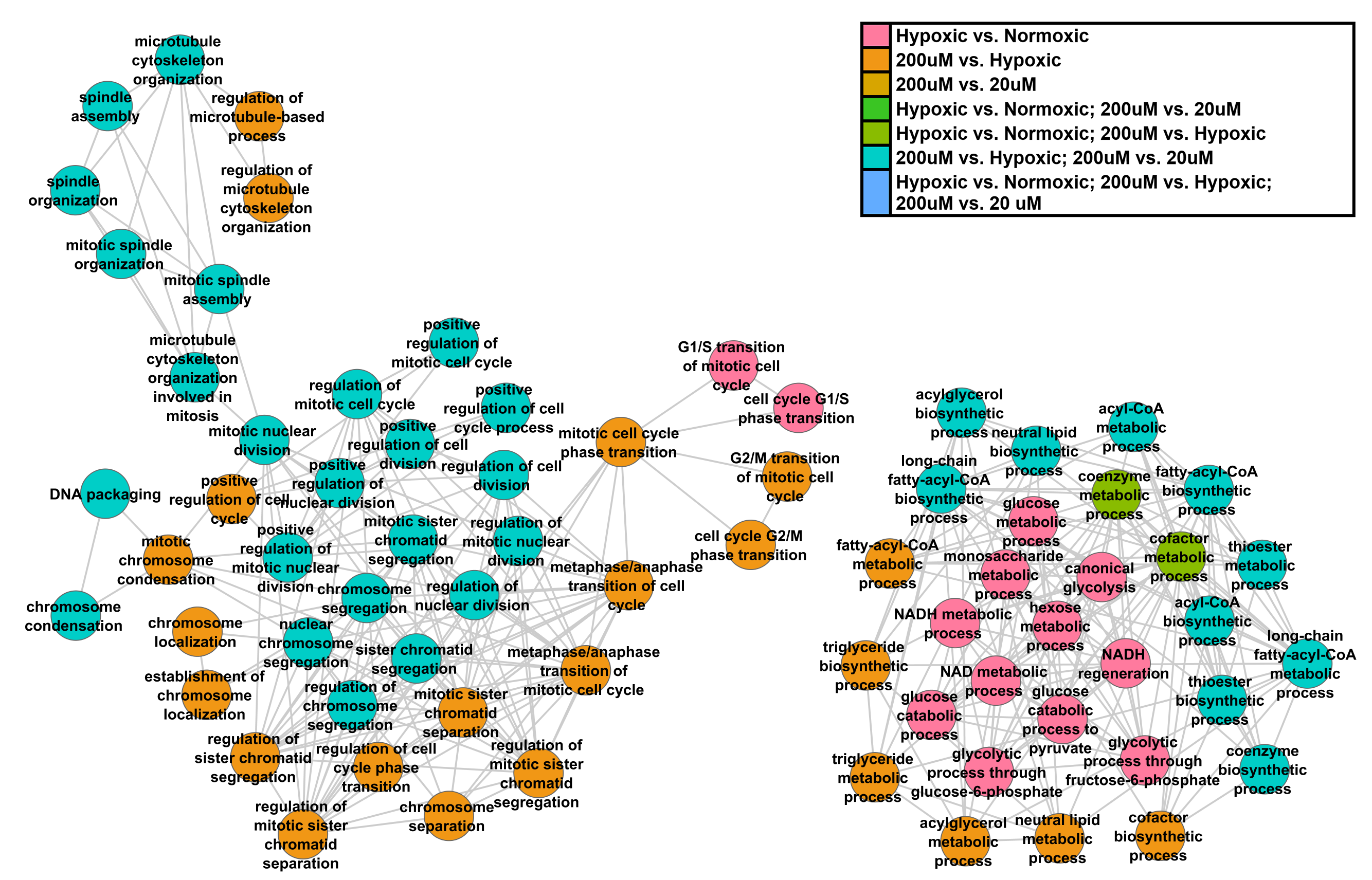

Supplement: S2 Fig — Nodes represent enriched annotations for DEGs. Edges represent relationship between annotations sharing high number of genes with pvalue cutoff 0.001 and edge weight greater than 0.90. Gene ontology (GO) enrichment analysis was performed using topGO with Fisher’s exact test. GO enrichment analysis was performed by the KBRIN Bioinformatics Core. (PNG) [file pone.0281396.s002.png]

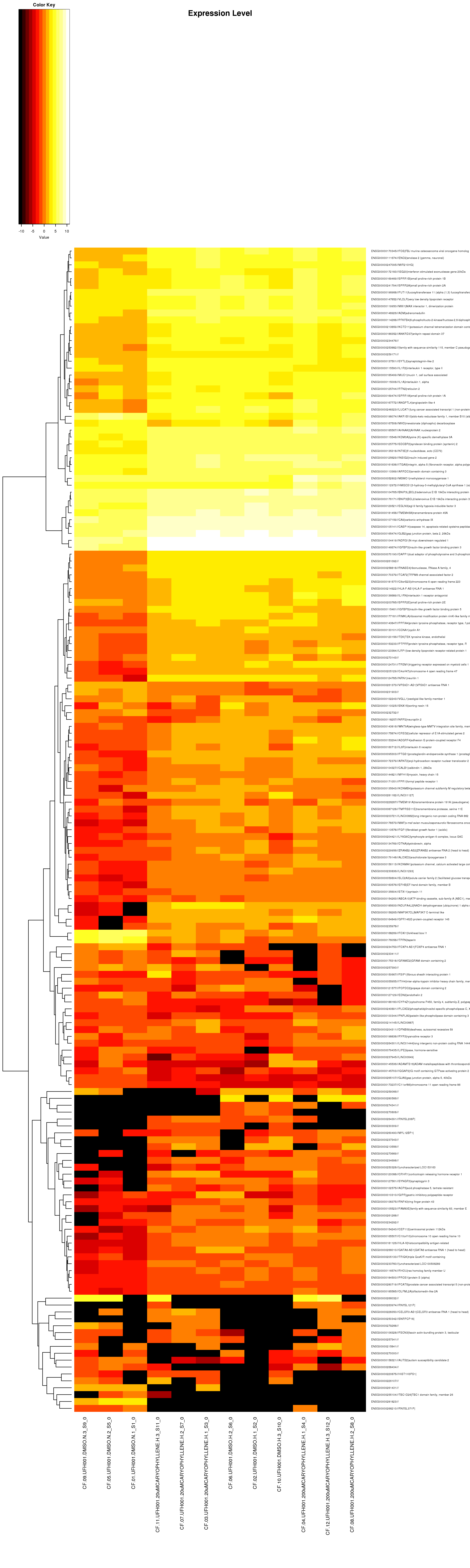

Supplement: S3 Fig — Genes showing a differential expression |Log2FC| ≥0 in at least one of the four pairwise comparisons (Normoxic v. Hypoxic, Hypoxic v. Hypoxic + 20 μM BCP, Hypoxic v. Hypoxic + 200 μM BCP, Hypoxic + 20 μM BCP v. Hypoxic + 200 μM BCP). Individual samples are clustered from left to right—Normoxic, Hypoxic + 20 μM BCP, Hypoxic, Hypoxic + 200 μM BCP. Heatmap analysis was performed by the KBRIN Bioinformatics Core. (PNG) [file pone.0281396.s003.png]
