# Supplementary material for: Effects of β-caryophyllene and oxygen availability on cholesterol and fatty acids in breast cancer cells
Source: PLoS One. 2023 Mar 9;18(3):e0281396. doi: 10.1371/journal.pone.0281396 (PMC9997903; doi:10.1371/journal.pone.0281396)
Supplement: S1 Raw images — Individual films for each of the proteins assessed. (PDF) [file pone.0281396.s004.pdf]

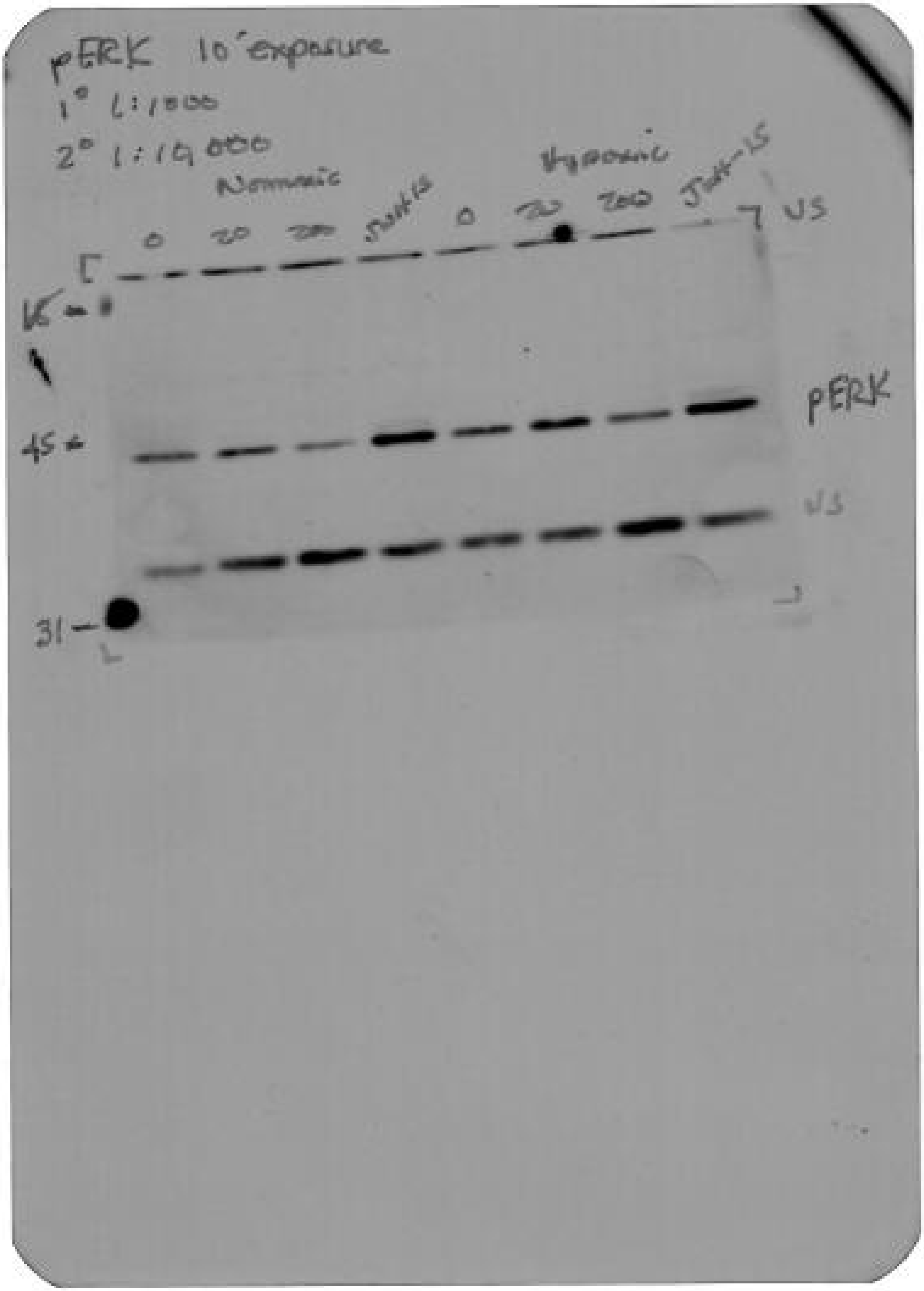
pERK 10' exposure
1° 1:1000
Normoxic
Hypoxic
0
20
200
0
20
200
VS
pERK
45
31

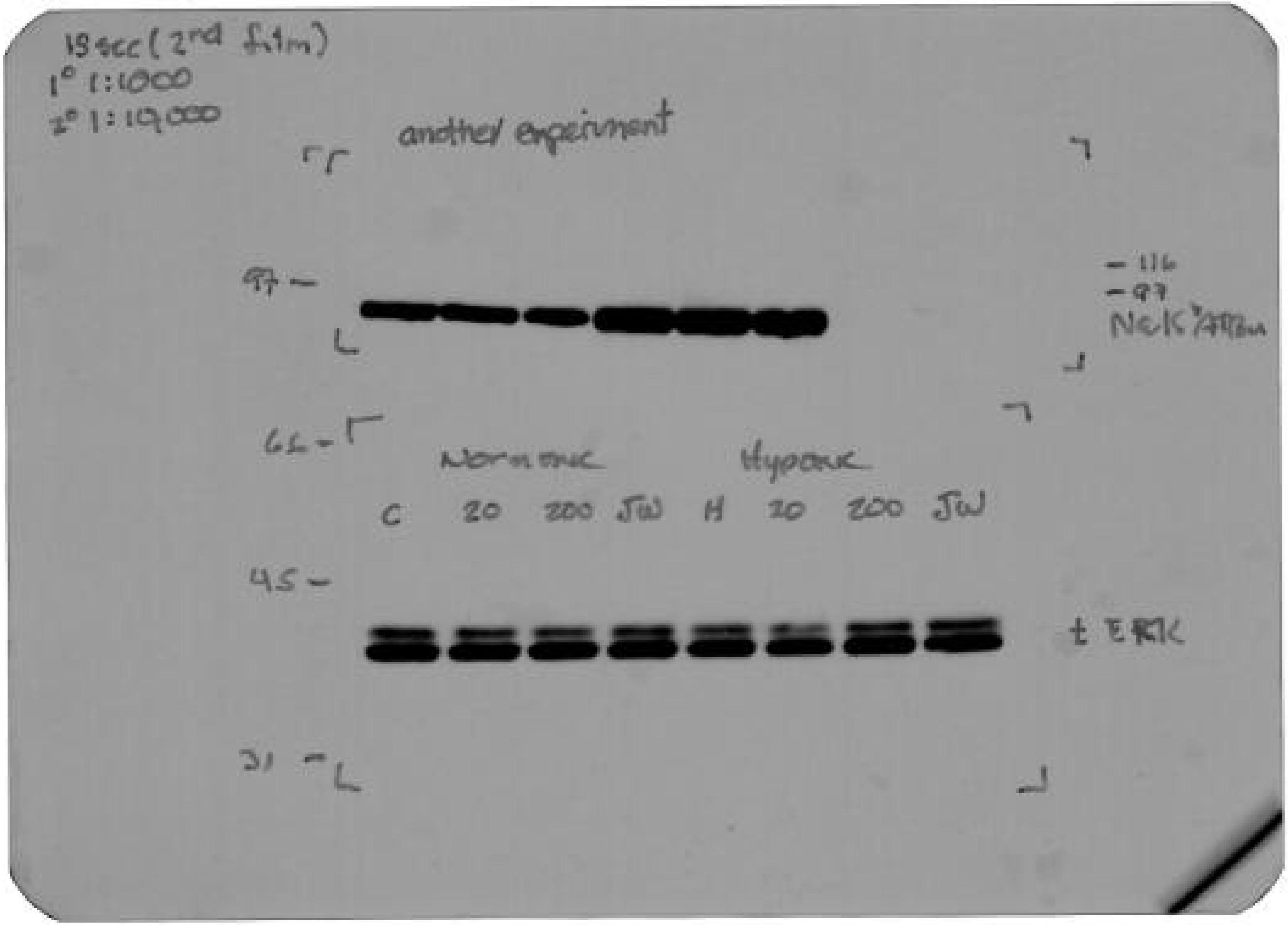
15 sec (2nd film)
1° 1:1000
2° 1:10,000
another experiment
97
116
97
66
Normoxic
Hypoxic
C 20 200 JW H 20 200 JW
45
t ERK
31

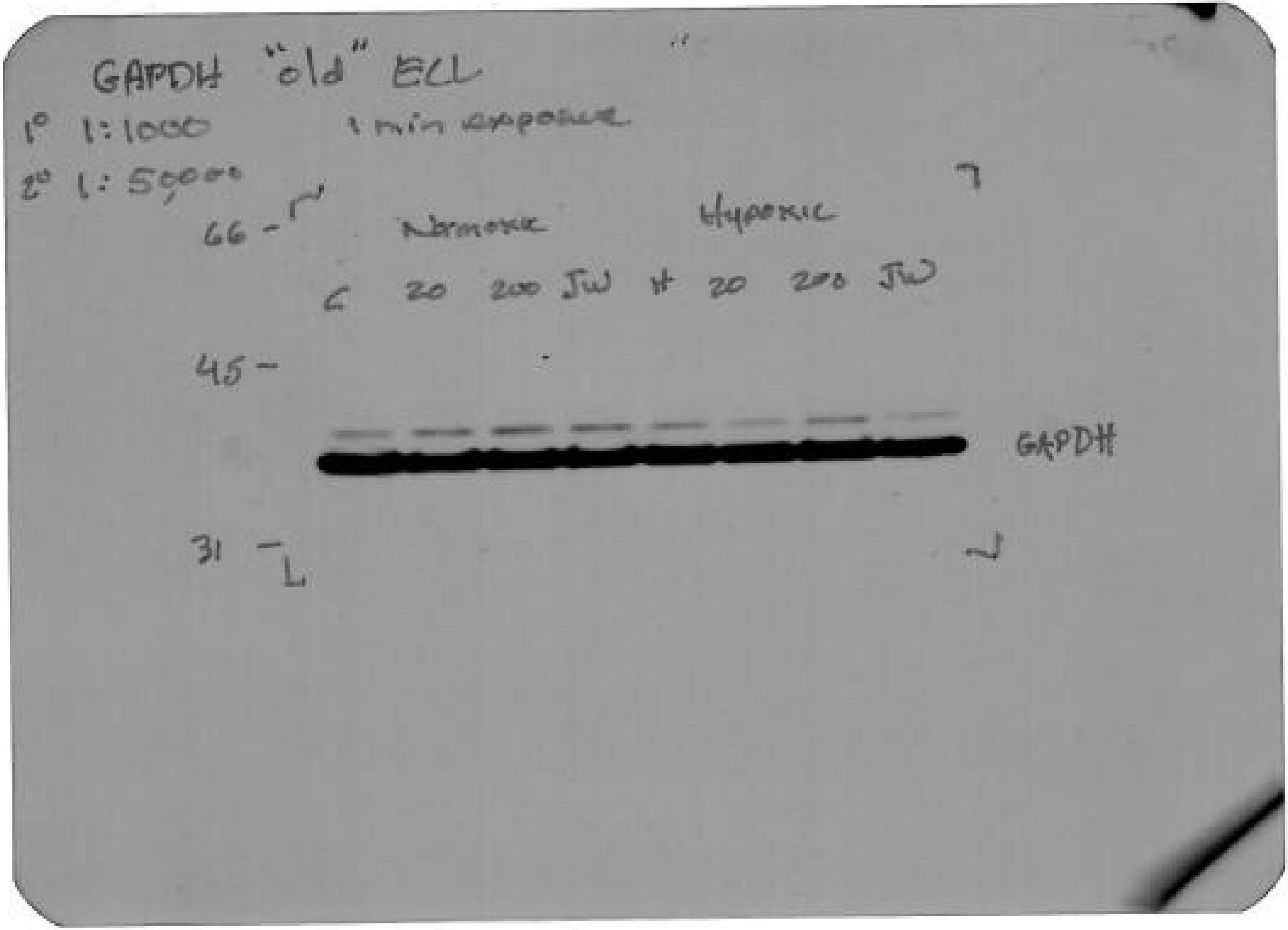
GAPDH "old" ECL
1° 1:1000
2° 1:50000
1 min exposure
66 -
Normoxic
Hypoxic
C 20 200 JW H 20 200 JW
45 -
GAPDH
31 -
